# Native mass spectrometry of membrane proteins reconstituted in peptidiscs

**DOI:** 10.1039/d5cb00236b

**Published:** 2025-10-22

**Authors:** Agrima Deedwania, Yi Wang, Carol V. Robinson, Jani R. Bolla

**Affiliations:** a Department of Biology, University of Oxford Oxford OX1 3RB UK carol.robinson@chem.ox.ac.uk jani.bolla@biology.ox.ac.uk; b Department of Chemistry, University of Oxford Oxford OX1 3QZ UK; c Kavli Institute for Nanoscience Discovery, University of Oxford Oxford OX1 3QU UK

## Abstract

Membrane proteins and lipids are essential for a wide range of cellular processes, making their structural characterisation essential for understanding biological function. However, the amphipathic nature of membrane proteins poses a significant challenge for traditional structural biology techniques. Membrane mimetics offer an alternative approach to studying membrane proteins in more native-like environments. Among them, peptidiscs have emerged as a promising tool for stabilising membrane proteins, allowing reconstitution from detergent micelles into a detergent-free, native-like environment that preserves structural integrity. While peptidiscs have shown utility in techniques such as mass photometry and cryo-EM, their compatibility with native mass spectrometry (MS) remains largely unexplored. In this study, we evaluate the feasibility of using peptidiscs for native MS analysis of membrane proteins and their complexes, focusing on the antibiotic resistance efflux pump AceI and the β-barrel assembly machinery (BAM complex). We reconstituted these proteins into peptidiscs using both ‘on-column’ and ‘on-bead’ assembly methods and assessed complex integrity and stability post-reconstitution using native MS. Our findings highlight the potential of peptidiscs as a tool for native MS-based structural characterisation of membrane protein and their assemblies.

## Abstract 2

Membrane proteins and lipids are essential for a wide range of cellular processes, making their structural characterisation essential for understanding biological function.

## Graphical abstract

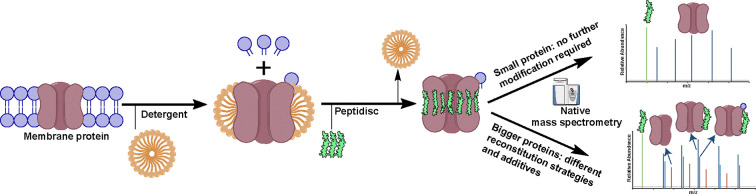

## Introduction

Membrane proteins and lipids play crucial biological and regulatory functions in a cell. They facilitate cell–cell signalling and control and regulate the import-export of chemicals, energy, and information across the cell membrane.^1,2^ Throughout these functions, membrane proteins interact with lipids, other proteins, and molecules in the cell and can change and regulate their structure, conformation, and function.^3,4^ Moreover, the mode of action of 60% of drugs involves membrane protein targets, and it has also been shown that they bind primarily to the transmembrane region.^1,5–7^ Hence, it is crucial to conduct structural studies of membrane proteins and their complexes to comprehend various cellular processes and develop effective therapeutics. In this regard, native mass spectrometry (MS) has emerged as an excellent means of studying membrane proteins and their complexes, as well as their interactions with lipids and small molecules in their folded states.^3,8–13^ Native MS also provides information on homogeneity, folding, modifications, subunit interactions, and the oligomeric state of the protein.^14^

Conventionally, detergents are used to solubilise and extract these membrane proteins from the lipid bilayer for biophysical/biochemical studies. These detergents shield the hydrophobic regions of the membrane protein from water, preventing aggregation and maintaining their solubility. However, during solubilisation, these detergents, if used in high concentrations, often remove lipids, perturb interactions and destabilise protein conformations.^1,6,15,16^ Only a small number of protein-lipid interactions are typically resolved in MS, which may not represent the native lipid environment.^1^ It is also often difficult to define the optimum solubilisation and purification conditions. As a consequence, there is a need for other molecules to mimic the dynamic lipid environment to understand the structure and other biological functions of these membrane proteins.^2^

Various membrane mimetics have been introduced as alternatives to detergents to protect the hydrophobic core of membrane proteins, shielding them from the aqueous environment, and avoiding aggregation.^2,17–19^ One approach is to reconstitute the detergent-solubilised protein into membrane mimetics such as bicelles, amphipols, liposomes, and nanodiscs. Detergent-free membrane mimetics, which are more native-like in nature, can also be used for solubilisation. These make use of synthetic polymers like SMA (styrene maleic acid) and DIBMA (di-isobutylene maleic acid) to form intact nanodiscs.^18,20^ Applications of native MS using nanodiscs have been limited since, and it is often challenging to release the protein due to strong interactions between the protein complexes and the nanodisc.^21–25^ It is hence an important task to optimise the protocol to eject various protein complexes from membrane mimetics using native MS.

Recently, peptidiscs have emerged as an alternative to existing membrane mimetics to reconstitute membrane proteins.^6,26,27^ Comprising small bi-helical peptide blocks, with optimal hydrophobic and hydrophilic properties, multiple copies wrap around the hydrophobic core of the membrane proteins and shield the protein from the aqueous environment.^28^ However, their applicability in native MS analysis remains highly limited. Here, we show that membrane proteins purified and reconstructed into peptidiscs can be analysed using native MS. Using this strategy, we compared two membrane proteins, AceI and BAM, in micelles and peptidiscs and show how they can be reconstituted differently and ejected.

## Results

### Reconstitution of AceI-Bril into peptidiscs

**Fig. 1:**
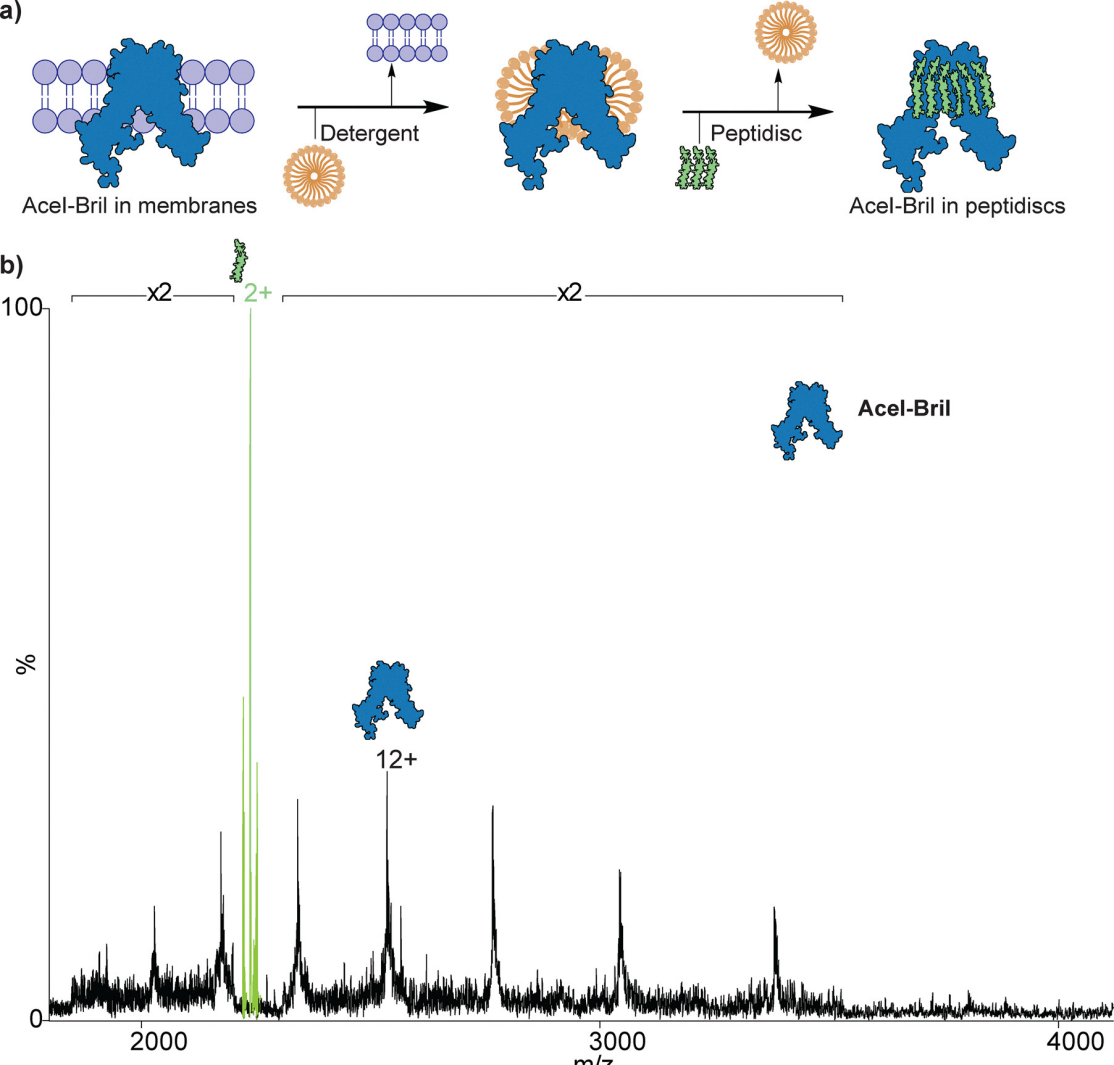
Reconstitution of AceI into peptidiscs and analysis by native MS. (A) Schematic of methodology used for reconstituting AceI-Bril into peptidiscs, (b) native MS analysis of AceI-Bril in peptidiscs. The spectrum shows the released peptide (green), and the other series corresponds to the intact AceI-Bril monomer (black).

To establish our methodology, we employed a small 4-transmembrane α-helical protein, AceI. We used an AceI-Bril construct for higher yields since previous studies have reported that the yield of non-modified AceI constructs is very low.^29^ We used this protein to establish our method since it is a relatively small 30 kDa membrane protein.^30^ After overexpressing this protein in *E. coli* and using a Ni-NTA column for purification of the protein in peptidisc using on-bead methodology, we applied SDS PAGE analysis (Fig. 1a and Fig. S1). Further purification of the reconstituted AceI in peptidiscs using size exclusion chromatography (SEC) revealed that all proteins eluted as aggregates in the void volume (Fig. S2), similar to the behaviour in detergent micelles. Hence, the protein, after affinity purification, was concentrated, buffer exchanged, and then used for MS analysis.

### Ejection of AceI-Bril from peptidiscs

To evaluate the compatibility of peptidiscs with native MS, we compared AceI-Bril samples prepared *via* two approaches: detergent-based solubilisation and peptidisc reconstitution. AceI-Bril was initially purified in DDM and subsequently buffer-exchanged into 200 mM ammonium acetate with 0.05% LDAO (pH 8.0) for native MS analysis (Fig. S3). In parallel, peptidisc-reconstituted AceI-Bril underwent buffer exchange into 200 mM ammonium acetate (pH 8.0). AceI-Bril exhibited a monomeric species when ejected from LDAO micelles, yielding an observed mass of 30 421 ± 0.5 Da, consistent with prior reports^30^ (Fig. S3). The peptidisc-reconstituted preparation similarly showed a protein mass of 30 418 ± 3 Da, confirming the structural integrity of monomeric AceI-Bril in both environments (Fig. 1b). A peak at 2237 *m/z* was observed as a +2 charge state, which was not present in the detergent micelle, suggesting that it corresponds to the peptidisc. The observed mass of peptidisc (4473 Da) also matched the expected mass (4474 Da). The energy required to eject the protein from the peptidisc (250 V) was higher than that required for removing detergent micelles (120 V).

### Reconstitution of the BAM complex using on-bead method

**Fig. 2:**
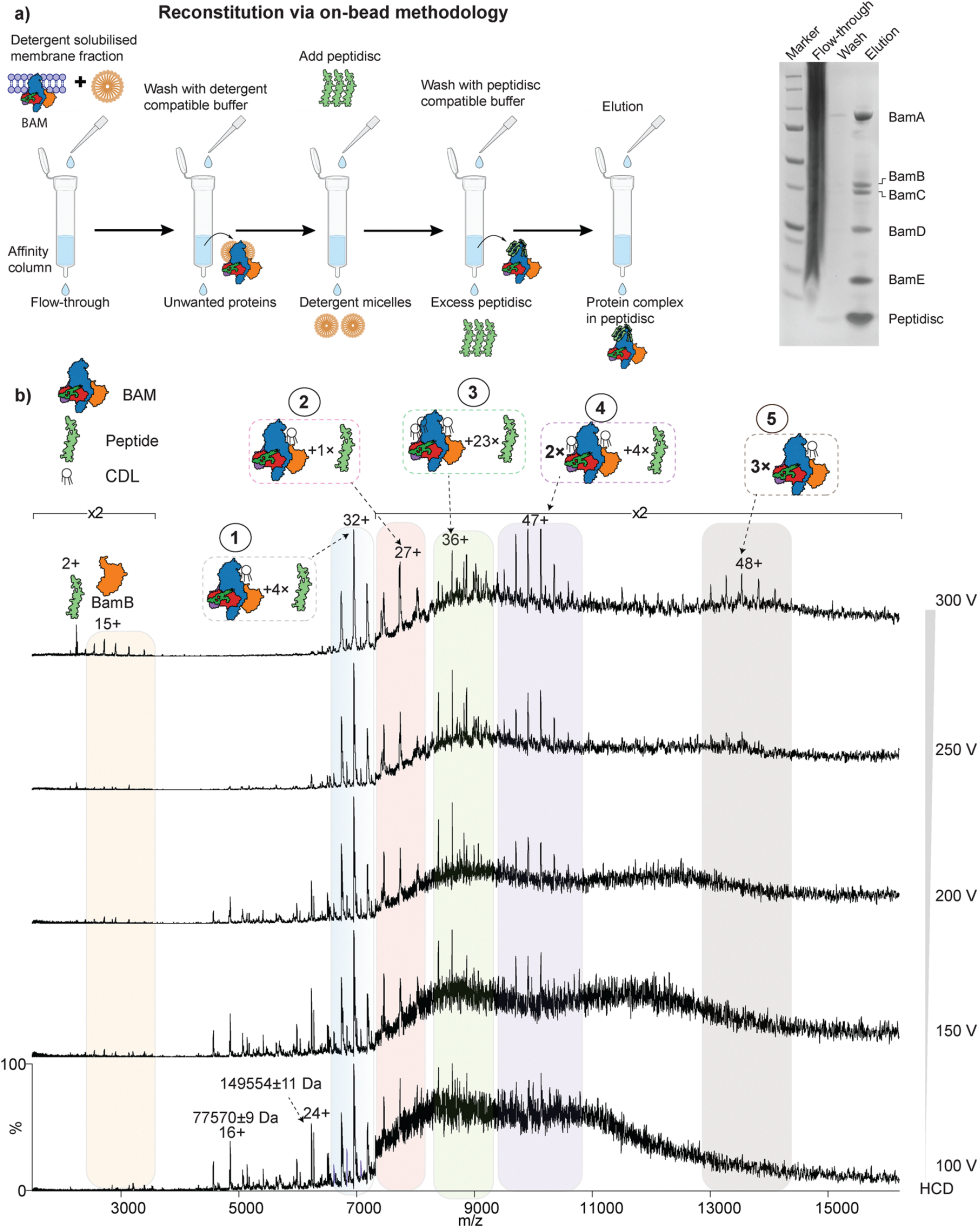
On-bead reconstitution of BAM complex and analysis by native MS. (a) schematic of the workflow used for reconstituting BAM complex into peptidiscs using on-bead method. SDS-PAGE gel analysis of the purification is shown on the right. (b) native mass spectra of on-bead reconstituted BAM complex acquired under increasing amounts of higher-energy collisional dissociation (HCD) energies. Peaks corresponding to various masses are observed, most of which can be assigned to BAM complexes associated with peptides and/or lipids in different combinations and oligomeric states. Several peaks (*e.g.*, 1, 2, and 5) could be confidently assigned, while others remain ambiguous due to possible overlapping combinations of lipids and peptides. #3 can also correspond to BAM with an additional BamB subunit (along with 14 peptides) that was assigned in a recent mass photometry study.^6^ At very high HCD energies, a distinct release of BamB and the peptide is observed. Notably, masses at approximately 77.5 kDa and 149.5 kDa could not be confidently assigned and may correspond to co-purified contaminants.

Inspired by the success of releasing monomeric AceI-Bril from peptidiscs, we next explored the potential for multi-subunit membrane proteins reconstituted into peptidiscs. Selecting the BAM complex, an assembly essential for outer membrane protein biogenesis, this β-barrel assembly machinery is composed of 5 subunits (BamA-E ∼ 203 kDa). This complex is known to co-purify with lipids (such as phosphatidylethanolamine, PE, and cardiolipin, CDL) and to dissociate in detergent micelles readily.^31–33^ We explored different reconstitution strategies, namely *via* on-bead and on-column peptidisc approaches. For the on-bead method, we incubated detergent-solubilised BAM with peptidisc peptides and resin-bound affinity tags to facilitate detergent removal (Fig. 2a). After extensive washing with peptidisc peptide, the complex was eluted using imidazole, yielding a preparation containing only the BAM complex embedded in peptidiscs. The data show that the complex can be purified to high purity with peptidisc peptide surrounding it (Fig. 2a).

### On-bead reconstituted BAM complex spectrum is very heterogeneous

Following buffer exchange into 200 mM ammonium acetate (pH 8.0), we recorded a native mass spectrum of the reconstituted BAM complex. A highly heterogeneous spectrum, with multiple overlapping charge states, was observed. To explore this spectral ambiguity, we increased the activation energy from 100 V to 300 V incrementally (Fig. 2b). This adjustment led to a notable reduction in background noise and significantly improved the resolution of spectral features. Under low energy conditions (100 V), in addition to smaller masses (∼77.5 kDa and ∼149.5 kDa), peaks can be observed consistent with the expected intact BAM complex (222 469 ± 12 kDa and 208 949 ± 5 Da). These series were assigned to the BAM complex with one lipid (CDL) associated with four and one peptide, respectively. As the energy is increased, increased resolution reveals BamB (40 690 ± 13 Da), as well as several other species (Fig. 2b). These species are tentatively assigned to BAM with several lipids and peptides or dimeric and trimeric BAM with additional peptides/lipids (Fig. 2b). A similar pattern was also observed when BAM was analysed in peptidiscs by mass photometry recently.^6^ These results suggest that the on-bead method may not be optimal for large, multimeric assemblies (Fig. 2b), and further optimisation is needed.

### On-column reconstitution of the BAM complex

**Fig. 3:**
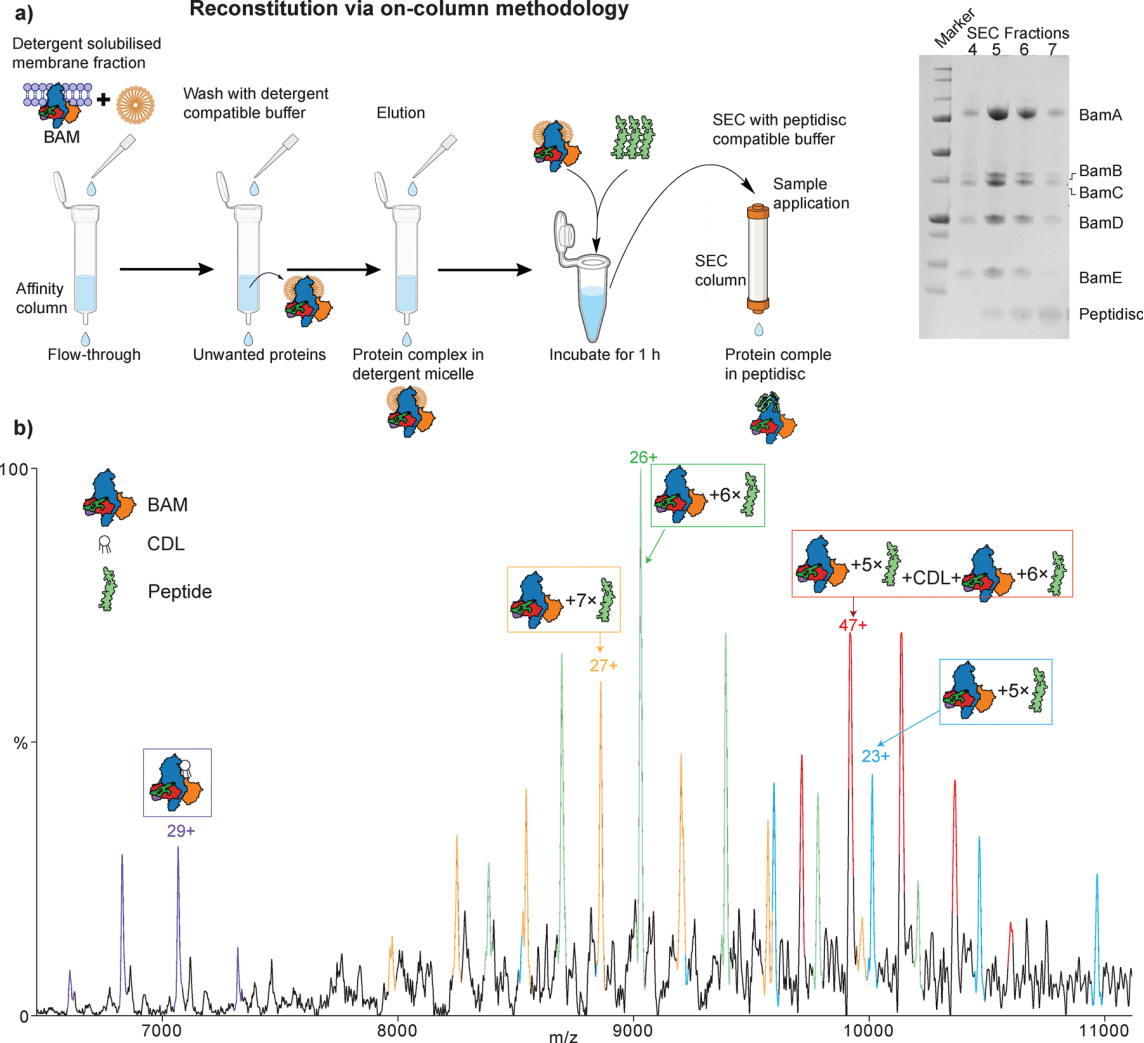
On-column reconstitution of Bam and native MS analysis. (a) schematic of the workflow used for reconstituting the BAM complex into peptidiscs using the on-column method. SDS-PAGE gel analysis of the purification is shown on the right. (b) native mass spectrum of on-column reconstituted BAM complex. Distinct charge state series are seen, all of which can be assigned confidently to BAM with several peptides.

We reasoned that the complexity in resolving the spectra above is due to co-purified excess lipids as well as contaminant proteins. As an alternative to this, we explore on-column reconstitution of BAM using SEC. For this, we first purified the BAM complex in DDM. We incubated it with peptidisc peptide (at 1 mg mL^−1^) in a 1 : 1 ratio at 4 °C with gentle shaking for 1 h, allowing initial scaffold association with the membrane protein. Following this pre-incubation, the mixture was subjected to SEC, facilitating a gradual transition from detergent micelles to peptidisc encapsulation (Fig. 3a).

The resulting SEC chromatograms (Fig. S4) revealed a distinct elution profile with multiple fractions: fraction 4: high-molecular-weight aggregates, likely misassembled or partially unfolded BAM complexes; fractions 5–6: successfully reconstituted BAM complex encapsulated within peptidiscs, eluting earlier due to their increased size and reduced diffusion; fraction 7: which appears just after the BAM-peptidisc peaks, potentially represents BAM-peptidisc complexes with less number of peptides or peptidisc-associated BAM subcomplexes and fraction 11: unbound peptidisc peptide, eluting later due to its small size and monomeric state. SDS-PAGE analysis shows that, along with peptidisc, all the subunits are present (Fig. 3a).

### On-column reconstituted Bam complex is ejected readily

To evaluate the compatibility of this preparation with native MS, we acquired MS data using similar methods described above. Compared to the highly heterogeneous spectra observed with on-bead preparations, the on-column sample yielded markedly improved spectral resolution and more discrete peak profiles. We observed multiple distinct charge state distributions corresponding to intact complexes bound to several peptides (Fig. 3b). Namely, we observed the peaks corresponding to the BAM complex bound to CDL (204 908 ± 25 Da). This lipid was previously known to co-purify and was proposed to play a functional role.^32,33^ Also observed was the BAM complex with five, six, and seven peptides (observed masses 230 241 ± 16 Da, 234 749 ± 23 Da and 239 184 ± 12 Da, respectively (Fig. 3b)). All of which can be readily resolved with high precision. A mass of 466 015 ± 15 Da was also observed and tentatively assigned to dimeric BAM with multiple peptides. The energy required to eject BAM from peptidiscs (200 V HCD and 150 V in-source trapping) is slightly higher than for BAM from detergent micelles (200 V HCD) (Fig. 3b and Fig. S5). Despite this high energy, we did not detect significant amounts of BAM subcomplexes as compared to BAM in detergent micelles, where we observed the presence of BamACDE and BamACDE_2_ (Fig. S5). These results highlight the potential of peptidiscs to enable native MS of large membrane complexes, without dissociation, provided reconstitution is optimised to minimise heterogeneity.

### Diluting into a detergent buffer facilitates the release of proteins from peptidiscs

**Fig. 4:**
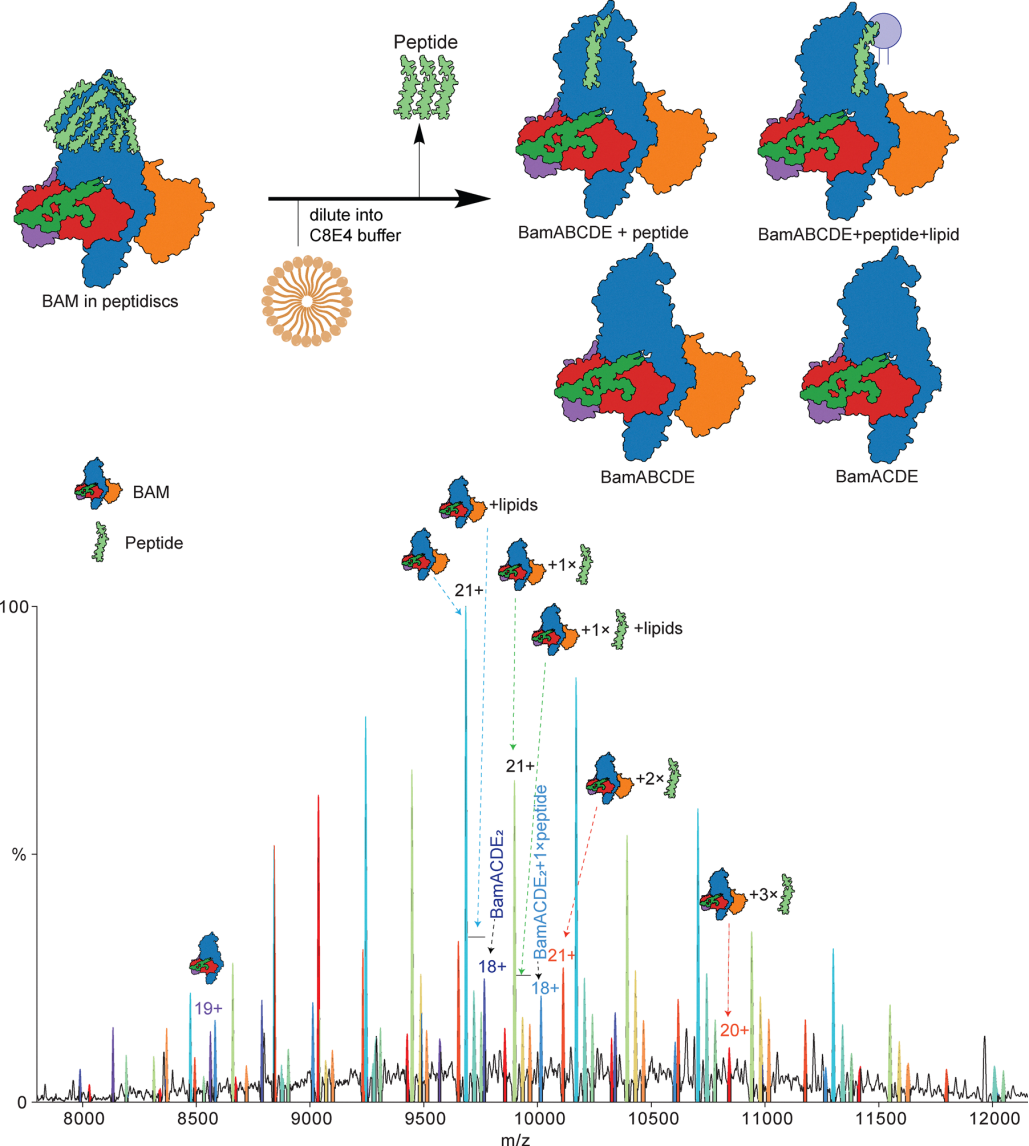
Effect of C8E4 on peptidiscs. Schematic is shown on top of possible products following dilution of the peptidisc preparation into C8E4 detergent. The resultant mass spectrum is shown below. In addition to subcomplexes, BAM with no lipids/peptides can be observed along with BAM with lipids and up to three peptides.

We next wondered if there is any way to facilitate the release of proteins/complexes from peptidiscs so that less dissociation energy is required. For this, we tested the effect of diluting the peptidisc preparation into C8E4, a detergent in which the BAM complex is stable and has been successfully used for structural and biochemical characterisation.^33,34^ Dilution of the peptidisc sample into buffers containing low concentrations of C8E4 significantly improved protein complex ejection efficiency and spectral resolution in native MS (Fig. 4 and Fig. S6 and S7). Under these conditions, BAM showed homogeneous mass spectra with reduced peptide adduct formation and clear lipid binding events. We also observed subcomplexes BamACDE and BamACDE_2_ with and without peptide (Fig. 4 and Fig. S7). This suggests that residual detergent can facilitate dissociation of the peptide scaffold during desolvation, aiding in the release of membrane proteins while preserving their native conformation.

## Discussion

In this study, we examined how peptidiscs can be used in native MS studies and assessed different reconstitution strategies for protein analysis. Our findings show that small membrane proteins, such as AceI, can be readily ejected without modification. In contrast, larger assemblies like the BAM complex required more extensive optimisation of reconstitution approaches and the inclusion of additives, such as small amounts of detergent.

Our comparison of two reconstitution strategies, on-bead *versus* on-column, highlights the importance of minimising sample heterogeneity for successful native MS analysis. The on-bead method produced highly heterogeneous spectra with masses that cannot be assigned with high confidence. In contrast, the on-column approach *via* SEC yielded cleaner, more defined spectra with unambiguous assignment of observed masses. These findings suggest that peptidisc-based sample preparation requires optimisation depending on the protein system and complexity of the target assembly.

Based on previous mass photometry measurements,^6^ approximately 15 peptidisc peptides are expected to associate with the BAM complex. In our native MS analyses, we observe a range starting from about four peptides, depending on the applied trapping energy and reconstitution method. Notably, the number of peptides associated with the BAM complex differed between the two reconstitution methods: on-column samples contained approximately five to seven peptides, compared with four peptides in the on-bead preparation (Fig. 2 and 3). This variation likely reflects differences in the starting material, the amount of co-purified lipid, and the overall efficiency of peptidisc reconstitution. We also found that diluting peptidisc samples into C8E4 detergent buffer significantly improved protein ejection efficiency and spectral resolution. This is probably because of partial loosening of the peptide scaffold, which facilitates the release of intact protein complexes.

One of the central challenges in native MS of membrane proteins is striking a balance between maintaining complex stability and achieving efficient desolvation and ejection. In our experience, smaller proteins such as AceI-Bril could be ejected from peptidiscs with moderate activation energy, while larger assemblies such as BAM required higher collisional energy. Importantly, even under these conditions, we did not observe extensive dissociation of BAM into subcomplexes, as typically seen in detergent-based preparations. These findings imply that peptidiscs stabilise multimeric assemblies, presumably by shielding vulnerable interfaces from solvent exposure or by offering lipid-like support that mimics the role of lipids.

Overall, our results demonstrate that peptidiscs offer a detergent-free alternative for stabilising membrane proteins within a lipid environment, making them particularly useful for downstream structural and functional studies where maintaining their native conformation is important. In contrast, detergent micelles can sometimes be too harsh, destabilising weaker complexes or removing bound lipids that are important for function. In our view, peptidiscs work well for maintaining complex integrity over longer periods, while detergents remain useful at earlier stages, such as for solubilising proteins or when rapid extraction is needed. A combined approach can often be most effective: adding small amounts of detergents to peptidisc reconstituted proteins prior to native MS. This reduces the energy required for protein ejection from the peptidisc, preserves key lipid interactions, and maintains overall complex stability. Compared with detergent-only environments, fewer subcomplexes tend to dissociate. In this way, native MS can be used not only to characterise reconstituted proteins but also to assess the quality of reconstitution, detect aggregation, and identify different stoichiometries within complex assemblies.

## Materials and methods

### Protein expression and membrane isolation

Plasmid for protein expression of *E. coli* Bam complex was obtained from Dr Harris Bernstein's group and *Acinetobacter baumannii* AceI-Bril (trunc-AceI-Bril construct) was obtained from our plasmid collection. Bril (also known as apocytochrome b562RIL) is a small, thermostable protein often fused to membrane proteins to improve expression, stability, crystallisation and structural analysis by cryo-EM. It's mainly a stabilising fusion tag. Both the plasmids are ampicillin resistant; hence the media was supplemented with 100 mg mL^−1^ Ampicillin for growth. Plasmids were first amplified by transforming into *E. coli* Stellar Competent Cells (Takara) and confirmed by Sanger sequencing. For protein expression, the plasmid for Bam complex was transformed into freshly prepared chemically competent *E. coli* BL21(DE3) cells, and the plasmid for AceI-Bril was transformed into chemically competent *E. coli* C43(DE3) cells (Cambridge Bioscience). Overnight preculture was grown at 37 °C by inoculating a freshly transformed colony in a 100 ml LB media. The preculture was diluted 1 : 100 into fresh 6 × 1L LB media and was grown at 37 °C until the OD_600_ reached 0.6. Cells were induced by the addition of 0.5 mM isopropyl-*b*-d-1-thiogalactopyranoside (IPTG), and the culture was grown for another 3 hours at 37 °C. Cells were pelleted using centrifugation with the Beckman JLA 8.1000 rotor for 15 minutes at 5000xg. The pellet was resuspended in 40 ml of 20 mM Tris-Cl pH 8.0, 150 mM NaCl buffer (Buffer A). Resuspended cells were supplemented with an EDTA-free protease inhibitor cocktail (Roche) and 1 mg mL^−1^ lysozyme (Thermo Scientific) and were homogenized and sonicated for cell lysis. The lysed cells were then centrifuged at 4 °C to separate the insoluble material using a JA 25.50 rotor at 20 000 × g, 30 min. The supernatant was collected and ultracentrifuged to isolate the membrane at 4 °C using a Beckman SW32Ti rotor at 200 000 × *g* for 1hour 30 min. Membranes were resuspended in 7 ml of 20 mM Tris-Cl pH 8, 150 mM NaCl buffer (Buffer A), and homogenised using a glass homogenizer.

### Peptidisc reconstitution

Resuspended membranes from 2 L of cell culture were solubilised in 20 ml buffer A supplemented with 2% *n*-dodecyl-β-d-maltopyranoside (DDM) (Anatrace) for 2 hours at 4 °C. The solution was then centrifuged at 20 000 × *g* for 30 min at 4 °C and filtered using 0.2 μm pore size to remove insoluble aggregates. The solubilized protein solution was then bound to a pre-equilibrated Ni-NTA column in buffer A supplemented with 0.03% DDM for 1 hour and was then allowed to pass using gravity flow. The column was then washed with 10-column volume of wash B: buffer A supplemented with 25 mM imidazole and 0.03% DDM and then with 5-column volumes of wash C: buffer A supplemented with 80 mM Imidazole and 0.03% DDM. At this stage, the protein was either eluted in a detergent micelle or was reconstituted in peptidisc. For elution in detergent micelle 25 ml of buffer A supplemented with 250 mM Imidazole and 0.03% DDM was used to pass through the column (this eluted protein was either used as protein in detergent micelle for downstream processes after SEC or was used for reconstitution in peptidisc using on-column method).

For reconstitution in peptidisc (Peptidisc Biotech) using on-bead, the resin beads after wash C were resuspended and homogenized in 50 ml of 1 mg mL^−1^ peptidisc solution in buffer A for 1 hour and were then passed through the gravity column. 10-Column volume of buffer A was then used to wash off the excess peptide. The reconstituted protein in peptidisc was then eluted using 25 ml of buffer A supplemented with 250 mM imidazole. Eluted protein was then analyzed on SDS-PAGE gel and peak fractions were pooled and concentrated using Vivaspin centrifugal concentrator. After concentration, for AceI-Bril protein, PD-10 desalting columns were used to change the protein to buffer A (also supplemented with 0.03% DDM for AceI in detergent micelle). For the Bam complex, the concentrated sample was further purified using size exclusion chromatography by Superdex 200 GL 10/300 column equilibrated in Buffer A (for the protein in the detergent micelle, the buffer was supplemented by 0.03% DDM). Eluted protein was then analyzed on SDS-PAGE gel, peak fractions were collected and concentrated. Samples were flash-frozen and kept at −80 °C until mass spectrometry analysis.

For reconstitution in peptidisc using on-column methodology, the purified protein in detergent micelle eluted through Ni NTA purification was incubated with peptidisc in 1 : 1 molar ration in cold condition with shaking for 1 h. The sample was further purified using size exclusion chromatography by Superdex 200 GL 10/300 column equilibrated in buffer A. Eluted protein was then analyzed on SDS-PAGE gel, peak fractions were collected and concentrated. Samples were flash-frozen and kept at −80 °C until mass spectrometry analysis.

### Native mass spectrometry

Using biospin-6 (BioRad) columns, proteins in peptidisc were buffer exchanged into 200 mM ammonium acetate pH 8.0, and proteins in detergent micelle were buffer exchanged into 200 mM ammonium acetate pH 8.0 supplemented with detergents 2× critical micelle concentration (0.05% LDAO (Lauryldimethylamine oxide) for AceI-Bril and 0.05% C8E4 for Bam Complex). Using gold-coated capillary needles, samples were introduced to the QExactive UHMR (Thermo Fisher Scientific) mass spectrometer. The instrument settings were as follows: capillary voltage 1.2 kV, S-lens RF 100%, quadrupole selection from 1000 to 20 000 *m/z* range, collisional activation in the HCD cell 100–300 V, trapping gas pressure setting 7.5, temperature 200 °C, and resolution of the instrument was set to 12 500. The noise level was set at 3 rather than the default value of 4.64. In-source dissociation was applied to a maximum of −150 V. The data was analysed using the Xcalibur 4.1 (Thermo Scientific) and UniDec^35^ software.

## Author contributions

Conceptualisation of project: C. V. R. and J. R. B.; methodology: A. D., Y. W., and J. R. B.; investigation: A. D., Y. W., and J. R. B.; funding acquisition: C. V. R. and J. R. B.; project administration: J. R. B.; supervision: J. R. B.; writing – original draft: A. D. and J. R. B.; writing – review and editing: A. D., Y. W., C. V. R., and J. R. B. All authors commented on the final version of the manuscript.

## Conflicts of interest

C. V. R. is a founder and consultant of OMass Therapeutics. All other authors have no competing interests.

## Supplementary Material

CB-007-D5CB00236B-s001

## Data Availability

The raw mass spectrometry data that support the findings of this study have been deposited in the Figshare database (https://doi.org/10.6084/m9.figshare.30305074). Supplementary information (SI) is available. See DOI: https://doi.org/10.1039/d5cb00236b.
