# Supplementary material for: Native mass spectrometry of membrane proteins reconstituted in peptidiscs
Source: RSC Chem Biol. 2025 Oct 22;7(1):120–8. doi: 10.1039/d5cb00236b (PMC12560048; doi:10.1039/d5cb00236b)
Supplement: CB-007-D5CB00236B-s001 [file CB-007-D5CB00236B-s001.pdf]

Supplementary Information (SI) for RSC Chemical Biology.
This journal is © The Royal Society of Chemistry 2025

Supplementary information

Native Mass spectrometry of membrane proteins reconstituted in peptidiscs

Agrima Deedwania[1], Yi Wang[1], Carol V Robinson[2,3#], Jani R Bolla[1,3#]

[1]*Department of Biology, University of Oxford, Oxford, OX1 3RB*

[2]*Department of Chemistry, University of Oxford, Oxford, OX1 3QZ*

[3]*Kavli Institute for Nanoscience Discovery, University of Oxford, Oxford, OX1 3QU*

[#]*Correspondence: Carol V Robinson (carol.robinson@chem.ox.ac.uk) and Jani R Bolla (jani.bolla@biology.ox.ac.uk)*

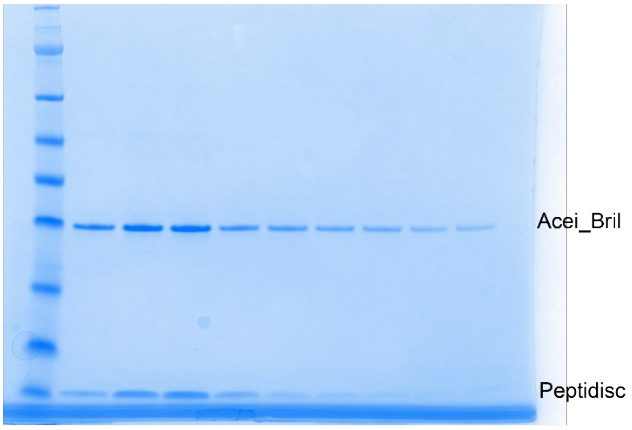

Figure S1: Successful reconstitution of AceI-Bril in peptidiscs. SDS-PAGE gel analysis of reconstituted AceI-Bril in peptidiscs. Clear bands for protein and peptide can be observed.

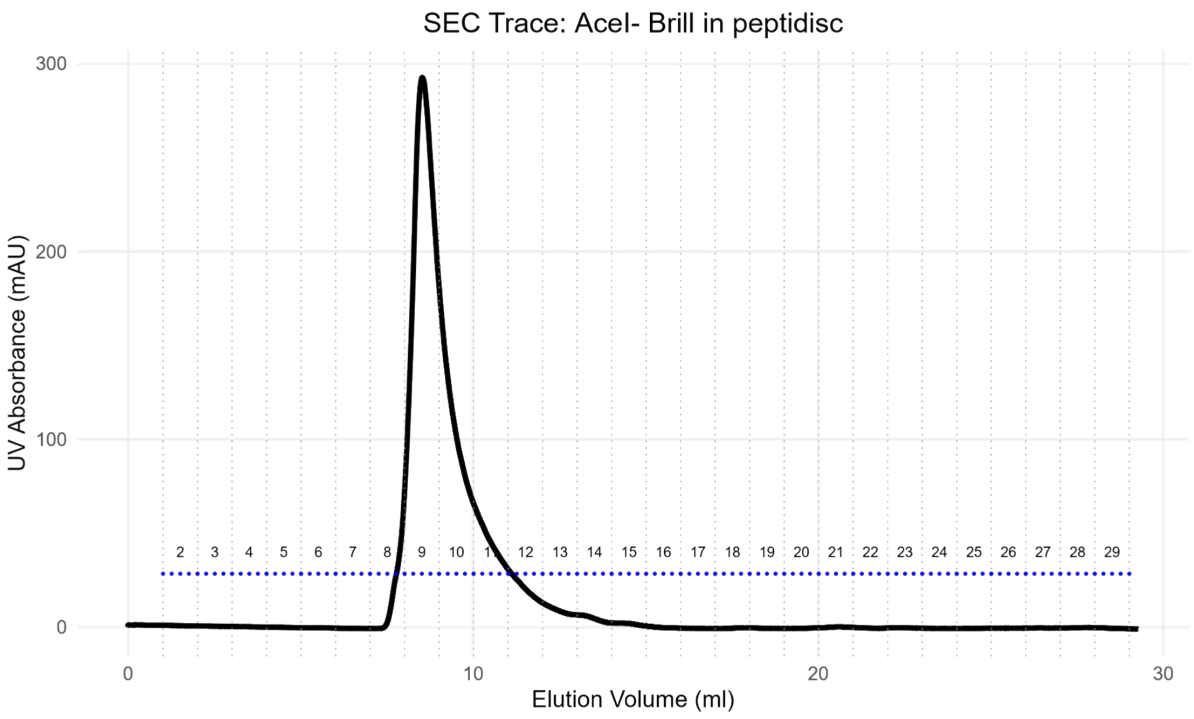

Figure S2: AceI-Bril in peptidiscs elutes at the void volume in SEC. SEC on AceI-Bril in peptidiscs shows that the protein is eluted as aggregates, with a strong peak appearing at the void volume. This behaviour is similar to that observed in detergent micelles before.

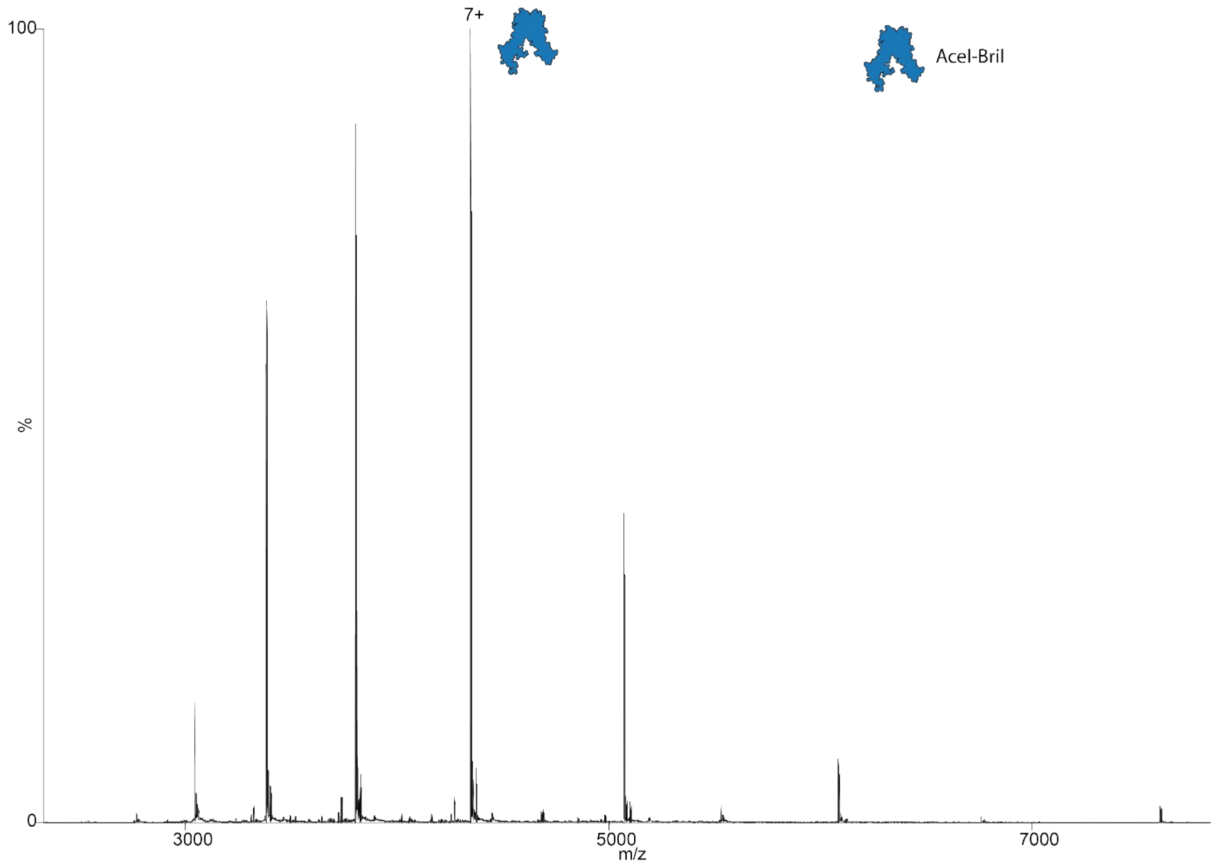

Figure S3: Native MS analysis of AceI-Bril in LDAO. Mass spectrum AceI-Bril purified in DDM, and buffer exchanged into LDAO. The data clearly show a single charge state series that corresponds to monomeric AceI-Bril.

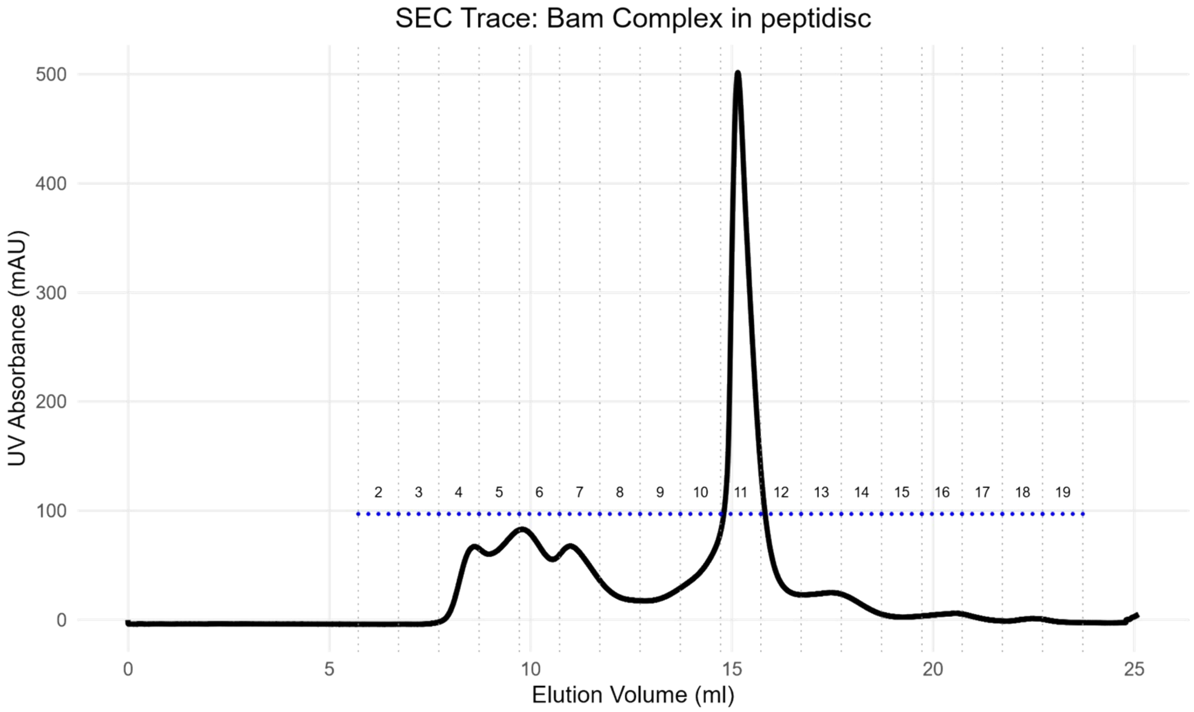

Figure S4: BAM complex in peptidiscs elutes in different fractions in SEC. SEC trace of BAM complex in peptidiscs shows several distinct peaks other than the peak at void volume. SDS-PAGE gel analysis shows the presence of Bam components in fractions mainly 5,6, and 7 (Figure 3a).

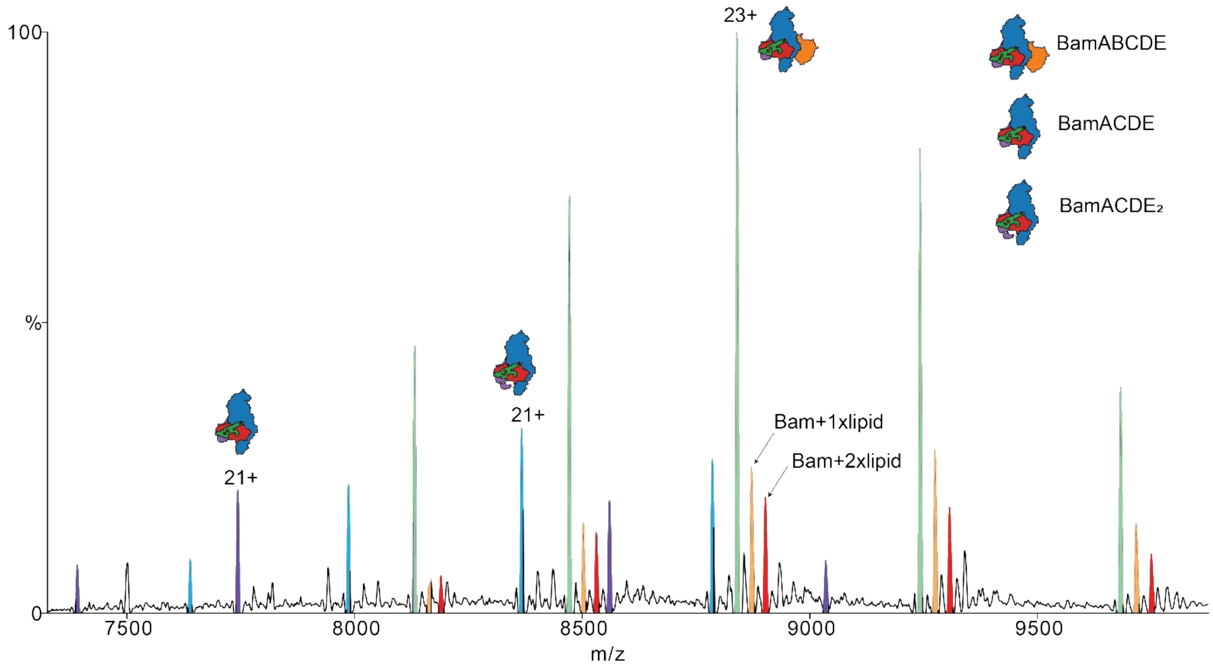

Figure S5: Native MS analysis of BAM complex in C8E4. Mass spectrum of BAM complex purified in DDM and analysed in C8E4. The spectrum displays distinct charge state series for intact BAM, both with and without lipids, as well as for subcomplexes BamACDE and $BamACDE_2$.

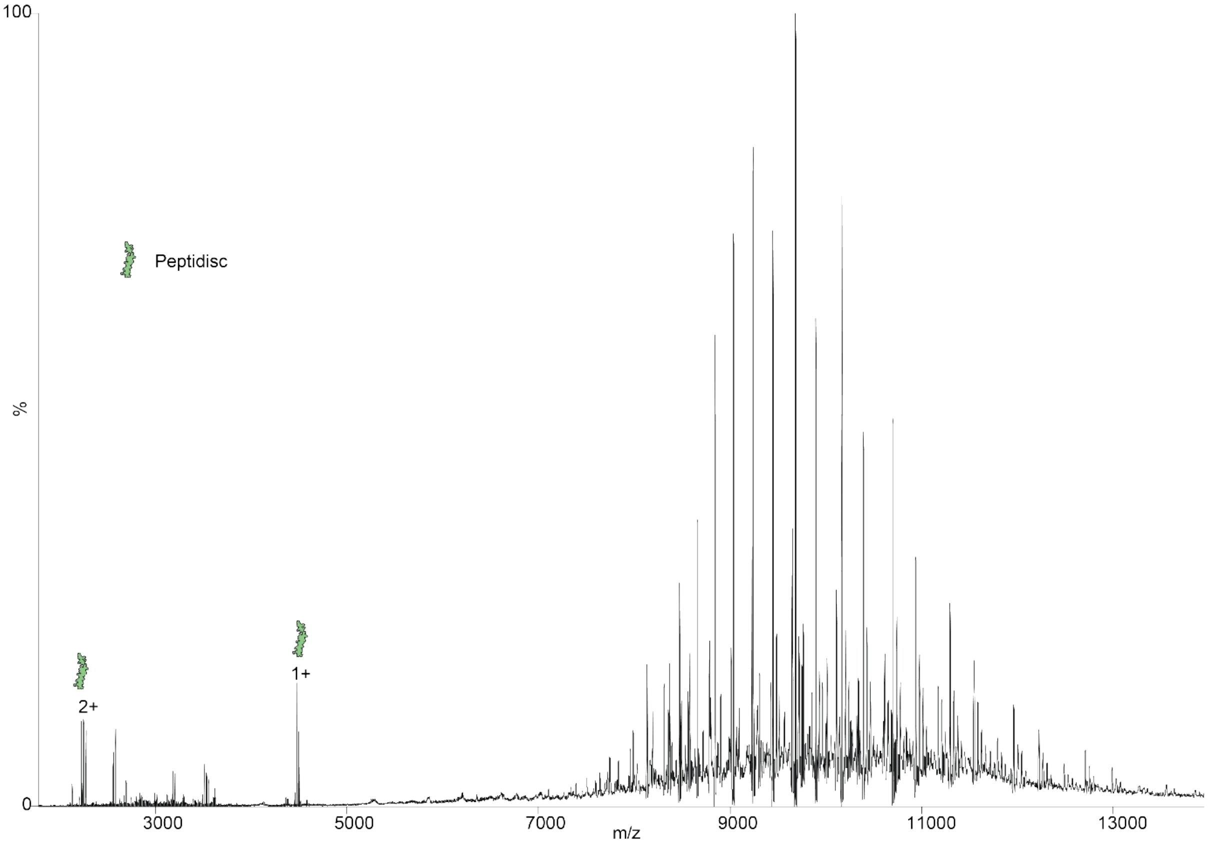

Figure S6: Full spectrum of detergent-diluted BAM peptidiscs sample. Mass spectrum of BAM complex in peptidiscs shows the release of peptides (as 2+ and 1+) upon dilution into C8E4 buffer. Complete annotation at a high m/z ratio is shown in Figure 4.

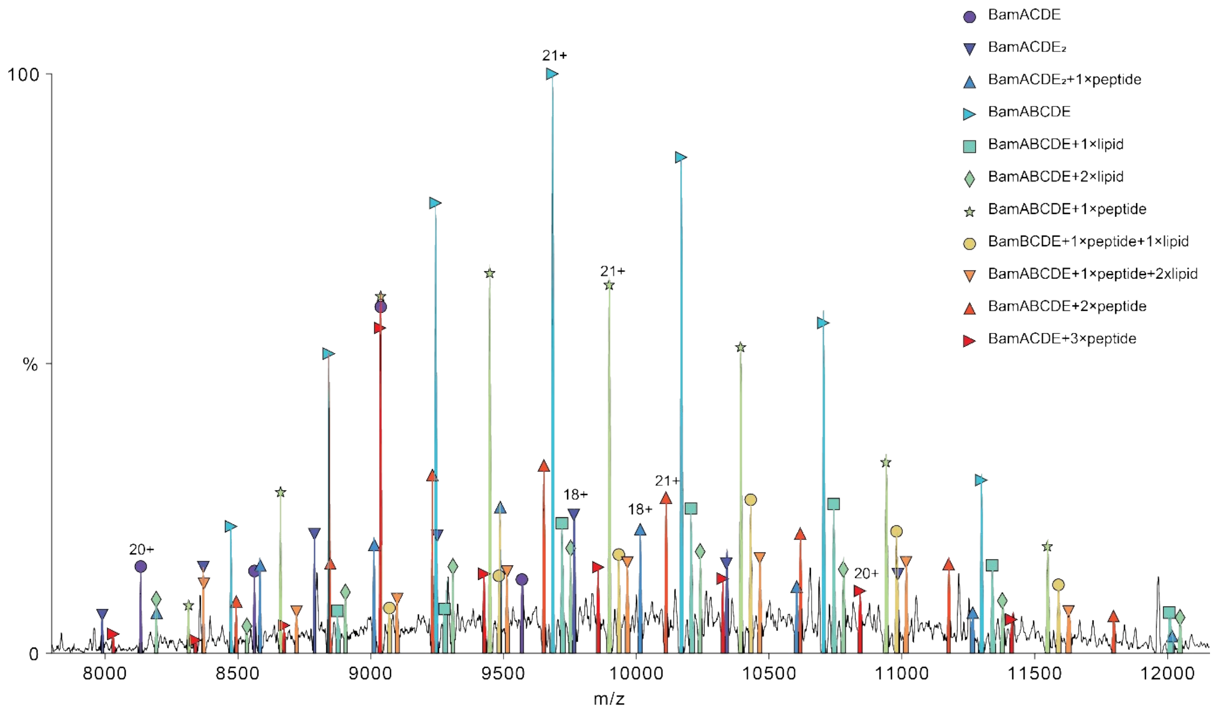

Figure S7: Assignment of detergent-diluted BAM peptidiscs spectrum. Complete assignment and annotation of the detergent-diluted BAM peptidiscs spectrum, shown in Figure 4.
